# Temperate Bacterial Viruses as Double-Edged Swords in Bacterial Warfare

**DOI:** 10.1371/journal.pone.0059043

**Published:** 2013-03-11

**Authors:** João Alves Gama, Ana Maria Reis, Iolanda Domingues, Helena Mendes-Soares, Ana Margarida Matos, Francisco Dionisio

**Affiliations:** 1 Instituto Gulbenkian de Ciência, Oeiras, Portugal; 2 Centro de Biologia Ambiental, Faculdade de Ciências da Universidade de Lisboa, Lisboa, Portugal; 3 Departamento de Biologia Vegetal, Faculdade de Ciências da Universidade de Lisboa, Lisboa, Portugal; 4 Center for Biodiversity, Functional and Integrative Genomics, Faculdade de Ciências da Universidade de Lisboa, Lisboa, Portugal; 5 Department of Biological Sciences and the Institute for Bioinformatics and Evolutionary Studies, University of Idaho, Moscow, Idaho, United States of America; University of Edinburgh, United Kingdom

## Abstract

It has been argued that bacterial cells may use their temperate viruses as biological weapons. For instance, a few bacterial cells among a population of lysogenic cells could release the virus and kill susceptible non-lysogenic competitors, while their clone mates would be immune. Because viruses replicate inside their victims upon infection, this process would amplify their number in the arena. Sometimes, however, temperate viruses spare recipient cells from death by establishing themselves in a dormant state inside cells. This phenomenon is called lysogenization and, for some viruses such as the λ virus, the probability of lysogenization increases with the multiplicity of infection. Therefore, the amplification of viruses leads to conflicting predictions about the efficacy of temperate viruses as biological weapons: amplification can increase the relative advantage of clone mates of lysogens but also the likelihood of saving susceptible cells from death, because the probability of lysogenization is higher. To test the usefulness of viruses as biological weapons, we performed competition experiments between lysogenic *Escherichia coli* cells carrying the λ virus and susceptible λ-free *E. coli* cells, either in a structured or unstructured habitat. In structured and sometimes in unstructured habitats, the λ virus qualitatively behaved as a “replicating toxin”. However, such toxic effect of λ viruses ceased after a few days of competition. This was due to the fact that many of initially susceptible cells became lysogenic. Massive lysogenization of susceptible cells occurred precisely under the conditions where the amplification of the virus was substantial. From then on, these cells and their descendants became immune to the λ virus. In conclusion, if at short term bacterial cells may use temperate viruses as biological weapons, after a few days only the classical view of temperate bacterial viruses as parasitic agents prevails.

## Introduction

Frederick W. Twort, in 1915, and Felix d’Hérelle, in 1917, discovered “a microbe that was “antagonistic” to bacteria and that resulted in their lysis” [1], [2], [3]. This kind of microorganism was later termed bacteriophage, which literally means bacteria eater. In 1940, with the advent of the electron microscope, the nature of this microorganism was revealed: it was a virus [4].

Bacteriophages (phages for short) are viruses that infect bacteria and can be generally classified as virulent or temperate (for a review see [5]). When a virulent phage infects a bacterial cell, it undergoes replication followed by the release of viral progeny, which results in host death. However, if the phage is temperate, two outcomes are possible after infection of a bacterium. Either there is production of viral progeny and host cell lysis similarly to what occurs for virulent viruses, or lysogeny occurs, meaning that the phage genome is stably incorporated as a prophage in the host cell (either integrated in the chromosome or as an episome). In the latter case, it is said that the host becomes lysogenic. This allows the phage genome to be replicated along with the host genome and consequently transmitted vertically to daughter cells. Some stressful conditions (such as UV radiation or thymine starvation) may induce the production of the viral progeny and, as a consequence, the host cell lysis [6].

Temperate bacteriophages can have several ecological roles. The conventional view is that they are either (i) parasites, because they exploit their host for reproduction, or (ii) predators, because bacteriophage replication and release usually kills the host. Lately, it has become evident that bacteriophages and bacterial cells may also establish a mutualistic relationship given that many phages code for virulence factors that will allow the bacteria to successfully infect hosts and expand their distribution [7], [8]. Another benefit of harbouring prophages, observed with *Escherichia coli* and several phages (λ, P1, P2 and Mu), is the fact that, during aerobic growth under conditions of continuous carbon source limitation, lysogenic strains have higher metabolic rate than non-lysogenic. Therefore, such lysogens reproduce faster than non-lysogenic strains [9], [10], [11], outcompeting them in environments where nutrients are scarce.

More recently, it has been proposed that pathogens and parasites in general may be useful to their hosts as biological weapons [12], [13], [14]. In particular bacteriophages may be useful to bacteria as antagonistic allelopathic entities [15]. However, this allelopathic agent is not a biochemical agent, but a “replicating agent” antagonistically affecting the growth, reproduction, or survival of other organisms (for reviews with a generalized view for the role of bacteriophages and other agents, see [16], [17]). In other words, lysogenic cells may use viral particles in a way similar to the use of toxins to kill non-lysogenic susceptible strains, thus possessing a fitness advantage over them. Moreover, the viruses may replicate inside bacteria, which die in process of virus release. Therefore, in contrast to typical toxins, the ability of phages to replicate inside their victims would give an additional advantage to the lysogenic bacterial population [14], [15], [18]. The amplification of phages inside their victims is particularly significant when only a small number of carriers of bacteriophages invade a susceptible bacterial population: within a few hours, the victims can substantially amplify the number of viral particles [15]. Alternatively, as mentioned before, temperate bacteriophages can establish a symbiotic relationship with the host. In this case, the cell is not killed and becomes immune to similar viruses.

Bacteriocins are proteins commonly coded in bacterial strains that kill other bacterial cells. In a similar way to what happens with bacteriophages, the antagonistic activity of bacteriocins is usually restricted to members of the same species; therefore, bacteriocins play an important role in competition between conspecifics [19], [20]. In this paper we sometimes give examples concerning colicins, which, by definition, are bacteriocins produced by *E. coli* (for a review on bacteriocins, see [21], and for a review on colicins, see [22]).

The colicin operon includes genes that code for the toxin itself and for immunity to this toxin. The operon of some colicin types also codes for a lysin. This lysin allows the producer to release the toxin to the environment, despite causing its own death. Therefore, and for similar reasons, the release of these colicins or of bacteriophages is costly to the cell that produces the toxin or the virus, respectively. However, clone mates of the producer are protected from its killing effect because they have the immunity gene. In other words, the antagonistic effect of bacteriocins (or of bacteriophages) is only directed to genetically distinct individuals [23], [24], [25].

Antagonistic interactions mediated by colicins can be viewed as spiteful interactions [26], [27], [28], [29], [30]. Given that bacteriocins kill susceptible bacteria whereas clone mates of producers are immune to it, the bacteriocin producer and its victims are negatively related [27], [28]. Gardner and West (2004) noted that there are two main elements involved in bacteriocin-mediated competition ([31]; see also ref. [32]): (i) the spatial scale at which competition for resources takes place; and (ii) the fraction of social partners that are clonal kin [29], [30]. .

In a structured habitat, competition is local. However, in unstructured habitats, all (or most) individuals in a population are social partners and competition is (almost) entirely global. Therefore, structured habitats offer better conditions for the evolution of spiteful behaviour [26], [27], [28], [29], [30].

There is a second reason why the effect of an antagonistic interaction like the one mediated by bacteriocin [23], [24], [33] or antibiotic [34] production depends on the structure of the habitat. In these habitats the killing of susceptible bacteria frees unused resources on the proximity of the cells coding for the toxin [24]. This is different from what happens in mass habitats, where freed resources are randomly distributed and equally available to all individuals in the population [24].

Both factors (spiteful interaction followed by competition for resources) can lead to the following effects of bacteriocins. In unstructured habitats, (e.g., liquid habitats where individuals affect equally the environment of every other individual), there is advantage of strains coding for bacteriocins if their frequency is above a certain threshold, otherwise bacteriocin production is disadvantageous. This was observed with the colicin E3 [24]. However, in structured habitats (where an individual has a stronger effect on neighbours) bacterial cells coding for colicin E3 are at an advantage even if initially rare [24].

As we have seen, temperate bacteriophages and bacteriocins share several properties. However, they are different in two fundamental aspects. On one hand, bacteriophages may replicate inside victims, and this may lead to an overall increase in the number of bacteriophages; this property confers an advantage to lysogens. On the other hand, temperate bacteriophages, once inside their new hosts, may establish themselves and confer immunity to similar bacteriophages; this property confers a disadvantage to the original lysogenic cells. Actually, genes coding for bacteriocins and immunity may also be transferred between bacterial cells [15], but one may expect plasmid transfer rate to be much lower [35], [36] than in the case of phages.

We thus ask: given that these two properties of temperate viruses originate opposing selective forces, which one prevails? And what is the role of habitat structure? To answer these questions, we study the effect of bacteriophages in structured and unstructured habitats and check the relative importance of virus amplification and lysogenization. We further compare our results concerning bacteriophages with previous results on the effect of bacteriocins [23], [24], [33], [37] and other toxins [34].

## Materials and Methods

### Bacterial Strains

In this study, we used *E. coli* MG1655 Δ*ara* λ^+^ streptomycin-resistant (lysogenic strain) (Lys Str^R^), *E. coli* MG1655 Δ*ara* rifampicin-resistant (susceptible to λ phage, phage-free strain) (Sus Rif^R^), *E. coli* MG1655 Δ*ara* λ^r^ rifampicin-resistant (phage-free but resistant to λ) (Res Rif^R^), *E. coli* MG1655 Δ*ara* λ^+^ rifampicin-resistant (lysogenic strain) (Lys Rif^R^), and *E. coli* MG1655 Δ*ara* streptomycin-resistant (susceptible to λ phage, phage-free strain) (Sus Str^R^).

The strain *E. coli* MG1655 Δ*ara* λ^+^ streptomycin-resistant was obtained from *E. coli* MG1655 Δ*ara* and λ phage as follows. First we obtained the lysogen and only then we isolated a spontaneous streptomycin-resistant clone. To obtain the *E. coli* MG1655 Δ*ara* λ^+^ strain we added 0.1 ml of an overnight culture of the *E. coli* MG1655 strain to a tube of molten LB soft agar (LB with 7.5 g/l of agar), mixed, and poured it onto a plate of agar (15 g/l). Then, we put 5 µl of a stock of the “Papa” strain of λ phage (from CBS-Knaw - The Netherlands Culture Collection of Bacteria – NCCB3442). After 15 minutes (to allow for polymerization of the agar), we incubated the plate at 37°C. The next day, we obtained a turbid plaque. The turbidity is caused by the growth of cells that are immune (lysogens) or resistant to λ phage. We then purified bacterial clones that are not affected by λ phage and tested these clones for resistance to λ_vir_ (NCCB 3467). If it is affected by λ_vir_, it is a lysogenic clone. A clone that is neither affected by λ nor by λ_vir_ is resistant. So, we isolated a clone that is resistant to λ phage but susceptible to λ_vir_. Then we isolated a spontaneous streptomycin-resistant clone from this lysogenic clone by plating 0.1 ml of an overnight culture of *E. coli* MG1655 Δ*ara* λ^+^ in a LB-agar plate with streptomycin (100 µg/ml) and incubated overnight (37°C). After inoculation, we streaked one of the clones in a similar plate and incubated overnight (37°C). The previous step was repeated once more. Then a single clone was stored at −20°C.

The rifampicin-resistant *E. coli* MG1655 Δ*ara* strain was isolated by plating 0.1 ml of an overnight culture of *E. coli* MG1655 Δ*ara* on a LB-agar plate with rifampicin (100 µg/ml) and incubated overnight (37°C). The next two days we proceeded as explained before for the streptomycin-resistant strain.

The λ^r^ rifampicin-resistant *E. coli* MG1655 Δ*ara* was obtained as described for the lysogenic strain, except that we added 5 µl of a stock of λ_vir_ phage (instead of λ phage). As previously explained, a resistant *E. coli* can grow in the presence of this virulent phage (but neither susceptible nor lysogenic *E. coli* cells can grow in its presence).

Preparation of strains with reversed markers: the strain *E. coli* MG1655 λ^+^ rifampicin-resistant was obtained from *E. coli* MG1655 and λ phage as follows. We used the spontaneous rifampicin-resistant clone mentioned above (where it was used as susceptible λ-free strain). To obtain a lysogenic rifampicin-resistant *E. coli* MG1655 strain we proceeded as described above for the lysogenic streptomycin-resistant strain. As explained in the beginning of this section, the strain *E. coli* MG1655 Δ*ara* λ^+^ streptomycin-resistant was obtained first by obtaining the lysogen and only then we isolated a spontaneous streptomycin-resistant clone. Therefore, to obtain strains with reverted markers, we proceeded as follows. To obtain a λ-free streptomycin-resistant clone isogenic to the streptomycin-resistant *E. coli* MG1655 λ^+^ strain, we sequenced the mutation in the *rpsL* gene in that streptomycin-resistant lysogenic strain. Then, we isolated eighteen spontaneous streptomycin-resistant clones from the wild-type *E. coli* MG1655 strain and sequenced the *rpsL* gene in all of them. To obtain the streptomycin-resistant clones we plated 0.1 ml of each of the eighteen overnight cultures of *E. coli* MG1655 on a LB-agar plate with streptomycin (100 µg/ml) and incubated overnight (37°C). The next two days we isolated spontaneous resistant clones as explained above for the rifampicin-resistant strain and for the lysogenic streptomycin-resistant clone.

PCR amplification of the *rpsl* gene in each clone was performed with 2 µl of pure bacteria culture added to a 25 µl PCR mixture reaction with 0.2 mM of each deoxyribonucleotide triphosphate (dNTP), 1.25 µM of each primer [38], 1 U of Taq polymerase, 1X reaction buffer and 4 mM of MgCl_2_ (NZYTech company). The temperature profile consists of an initial denaturizing step at 95°C (10 min), followed by 35 cycles of 95°C (1 min), annealing temperature of 64°C (1 min), 72°C (1 min), and a final extension step at 72°C (10 min). The amplification products were visualized in an agarose gel (2%) stained with Red Safe (iNtRON Biotechnology). The PCR product was extracted from the agarose gel with GFX PCR DNA and Gel Band Purification Kit (GE Healthcare) and sequenced by Sanger sequencing method by the StabVida Company.

### Measurement of Growth Rates of the Strains Lys Str^R^, Sus Rif^R^, Res Rif^R^

We placed 10 µl of a pre-culture grown overnight at 37°C with agitation (170 rpm), in 10 ml of Luria Broth (LB) medium and incubated at 37°C with agitation (170 rpm). We did this in triplicate for each strain: the streptomycin-resistant *E. coli* K12 MG1655 Δ*ara* λ^+^, the λ-free susceptible rifampicin-resistant *E. coli* K12 MG1655 Δ*ara* strain, and the λ-resistant (and λ-free) rifampicin-resistant *E. coli* K12 MG1655 Δ*ara* strain. Every 30 minutes, a sample of 500 µl was taken to measure the optical density at 670 nm. Growth rates were determined by linear regression. One-Way ANOVA analysis was performed to compare the growth rates of the different strains.

### Fitness of Strains Lys Str^R^, Sus Str^R^, Lys Rif^R^ and Sus Rif^R^

The four strains used are Δara. To measure their relative fitness, we performed competition experiments, in LB medium at 37°C with agitation for 24 hours, against a reference strain (unaffected by phage lambda), S*taphylococcus aureus* (Ara^+^) in an approximate proportion of 1∶1. The strains were previously grown in liquid LB medium for 24 hours at 37°C with aeration. The values of the each strain were estimated by plating a dilution of the mixture in TA (short for tetrazolium and arabinose) agar. The relative fitness of each strain was calculated according to the expression: Log_2_[S(t)/S(0)]/Log_2_[R(t)/R(0)], where S(t) and R(t) are the final values of the strain assayed and the reference *S. aureus* strain respectively and S(0) and R(0) are the initial values of the same strains.

### Experimental Competitions

Competitions between lysogenic and susceptible strains were performed in structured and unstructured habitats. We initiated competitions at different initial ratios of lysogenic to susceptible strain (between 10^4^∶1 and 1∶10^4^). Competitions for each initial ratio were made in triplicate.

### Competitions in the Unstructured Habitat

We inoculated approximately 10^6^ total bacteria (at initial different ratios of lysogenic to susceptible cells) in 10 ml of liquid LB medium and incubated at 37°C with shaking (170 rpm). At 24 hours intervals for five days, the cultures were diluted 1000 fold in liquid LB medium and incubated again at 37°C and 170 rpm.

On the first, third and fifth days of competition, we screened for the densities of λ phages, of lysogenic cells, susceptible cells, susceptible cells that became resistant to λ phage (i.e., cells with the same marker as susceptible cells but that are no more affected by λ neither by λ_vir_), and susceptible cells that became lysogenic (i.e., cells with the same marker as susceptible cells that are no more affected by λ phage but that are susceptible to λ_vir_ phage). For that, we performed appropriate dilutions of the mixtures and plated on LA supplemented with streptomycin (100 µg/ml) or rifampicin (100 µg/ml). Phages were collected through filtration (0.22 µm) and its titer was determined (see bellow). The appearance of lysogenic or resistant individuals of the susceptible population was screened by streaking at least 30 colonies across LA plates containing λ or λ_vir_ phages along one diameter of the Petri dish. Colonies that do not grow when in contact with both phages are susceptible. Colonies that are able to grow in the presence of both phages are λ-resistant. A bacterial cell is lysogenic if its growth is inhibited by λ_vir_ but not by λ phages.

### Competitions in the Structured Habitat

We inoculated approximately 3×10^6^ total bacteria (at initial different ratios of lysogenic to susceptible cells) in 3 ml of molten LB top agar (3.75 g/l), poured the mixture on a Petri dish containing a basal layer of agar (15 g/l) [24] and incubated at 37°C. After 24 hours, the upper layer was scraped into 12 ml of MgSO_4_ 10^−2^ M, well mixed (agitated on vortex for 3 minutes), and diluted tenfold. We then inoculated 200 µl of the suspension in three milliliters of LB top agar. This procedure was repeated for the next four days.

On the first, third and fifth days of competition, we screened for the densities of λ phages, of lysogenic cells, susceptible cells, susceptible cells that became resistant to λ phage, and susceptible cells that became lysogenic. For that, we performed as explained above for competitions unstructured habitat.

### Determination of Phage Titer

Phages were collected through filtration (0.22 µm) and the titer was determined by spotting 10 µl of each serial dilution of the phage (spot test) on the surface of solidified lawns of *E. coli* MG1655 Δara Rif^R^ susceptible to the λ phage. After incubating at 37°C for a day, the number of phage plaques was determined. The bacterial lawn was prepared by pouring 3 ml of molten LB top agar (3.75 g/l), inoculated with 100 µl of an overnight bacterial culture, onto a Petri dish containing a basal layer of agar (15 g/l). Then, phage plaques were counted.

### Control Competitions

In order to isolate the effect of λ-phage in competition experiments, we performed further competitions, this time between lysogenic and resistant strains. The initial ratios lysogenic:resistant were 1∶10^6^, 1∶10^5^,1∶10^4^, …,10^6^∶1. The ratios of these competitions were measured after 24 and 48 hours. Competitions for each initial ratio were made in triplicate. Details about this procedure, including a mathematical model, are given in the Material and Methods S1.

### Measurement of the Density of Free λ Phage in Pure Cultures of Lysogenic Cells

We measured the density of free λ phage in pure cultures of lysogenic cells, both at unstructured and structured habitats as follows. Unstructured habitat: we inoculated approximately 10^6^ total bacteria in 10 ml of liquid LB medium. After 24 hours of incubation at 37°C with shaking (170 rpm), phages were collected through filtration (0.22 µm) and the titer was determined using the spot-test method. Structured habitat: we inoculated approximately 3×10^6^ total bacteria in 3 ml of molten LB top agar (3.75 g/l). This was then poured on a Petri dish containing a basal layer of agar (15 g/l) and incubated at 37°C. After 24 hours, the upper layer was scraped into 12 ml of MgSO_4_ 10^−2 ^M and well mixed. Phages were recovered by filtration and its titer was determined.

## Results

The generation times of the lysogenic streptomycin-resistant strain (mean ±2×Standard Error = 23.40±1.29 min), of the λ-susceptible rifampicin-resistant strain (24.51±0.79 min) and of the λ-resistant rifampicin-resistant strain (23.69±0.67 min) are not significantly different from each other (one-way ANOVA, d.f.(2,6), p = 0.30). The λ-resistant strain was included in this comparison because it has previously been shown that some mutations conferring λ-resistance may change bacterial growth rate [39]–[40].

**Figure 1:**
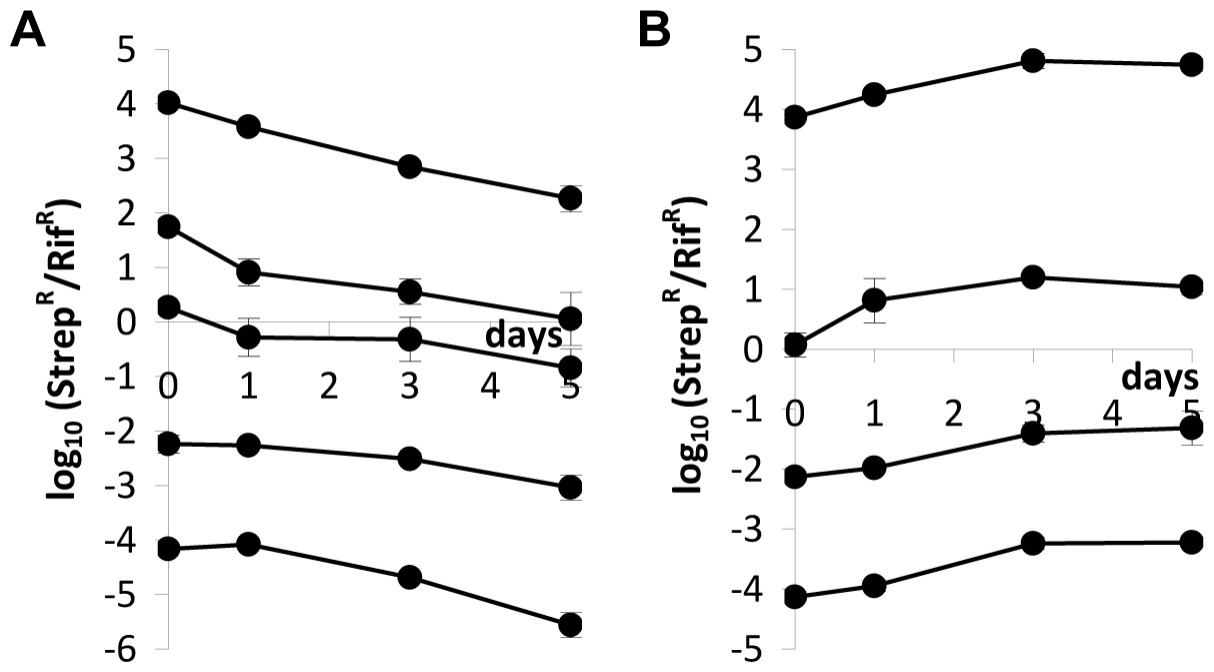
Logarithm of the ratio of streptomycin-resistant to rifampicin-resistant strains during competitions (A) in unstructured and (B) in structured environments. In the beginning of competitions, streptomycin-resistant cells were lysogenic and rifampicin-resistant cells were susceptible to λ phage. In the unstructured habitat, initial ratios were (lysogens:susceptibles) 1∶10^4^, 1∶10^2^, 1∶1, 10^2^∶1, and 10^4^∶1. In the structured habitat, initial ratios were 1∶10^4^, 1∶10^2^, 1∶1, and 10^4^∶1. Each data point represents the mean value of three independent competitions. Error bars represent twice the standard error of the mean (sometimes not seen because they are smaller than data points).

We then performed direct competitions in unstructured habitats (liquid LB medium) and in structured habitats (soft LB agar plates) between the lysogenic and susceptible strains starting at different initial ratios. Lysogenic cells were marked with streptomycin resistance and susceptible cells with rifampicin resistance. Colony forming units of each strain were counted on days 0, 1, 3 and 5 by plating cultures on LB-agar plates supplemented with streptomycin or rifampicin. Figure 1 shows the dynamics of the two bacterial populations in competition during five days.

In the unstructured habitat, the ratios of lysogenic to susceptible cells (streptomycin-resistant to rifampicin-resistant cells) decreased during the five days of competition (Figure 1A; linear regressions tell us that all lines have negative slopes; ANOVA, d.f. = (1,10); p<1.5×10^−3^ for all lines). Therefore, lysogenic cells are at a disadvantage at all initial ratios of lysogenic to susceptible cells (Figure 1A). Because susceptible cells (rifampicin-resistant) have the possibility to become lysogenic or resistant to the λ phage during the five days of competition, we looked for rifampicin-resistant cells that were also resistant or immune to the λ phage (see Materials and Methods) and found no sign of such cells.

**Figure 2:**
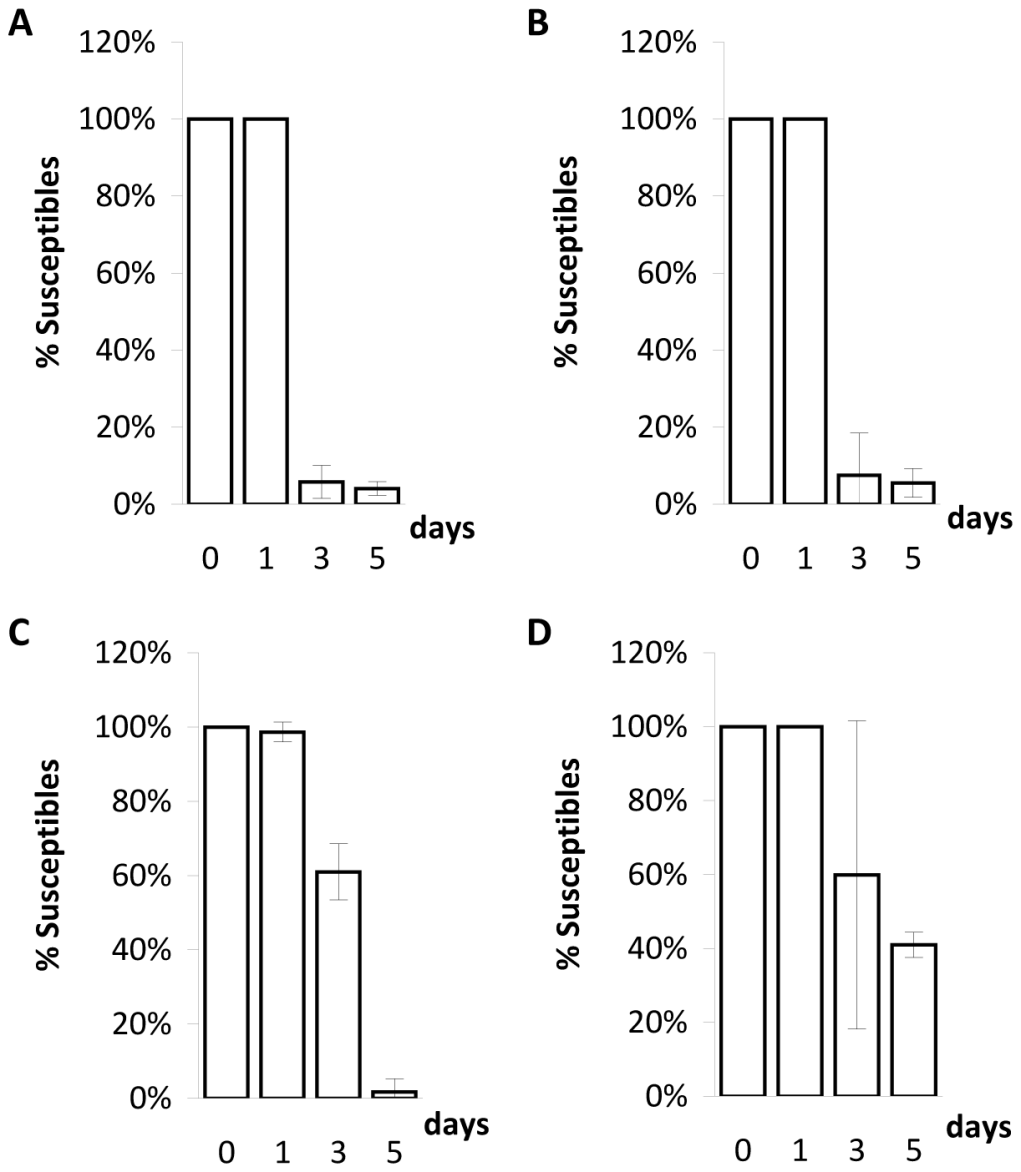
Proportion of susceptible cells among rifampicin-resistant cells during competitions in structured habitats. Each set of columns represent the mean of the percentage of rifampicin-resistant cells susceptible to λ phage at days 0, 1, 3 and 5. Error bars as in Figure 1. Ratios of lysogenic to susceptible cells as follows: A) ratio = 1∶10^4^; B) ratio = 1∶10^2^; C) ratio = 1∶1; D) ratio = 10^4^∶1.

**Figure 3:**
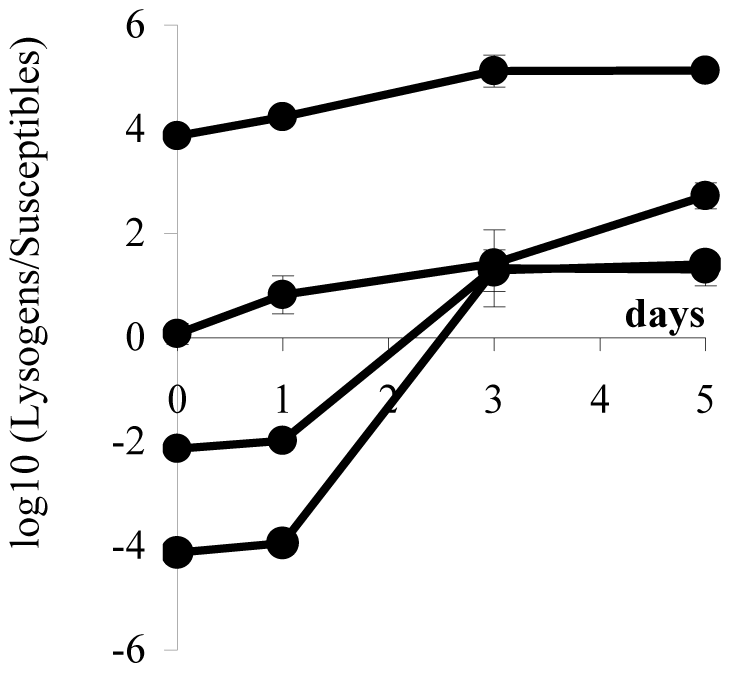
Logarithm of the ratio of lysogenic to susceptible bacteria during competitions in structured environments. Initial ratios were 1∶10^4^, 1∶10^−2^, 1∶1, and 10^4^∶1. Lysogenic cells include original lysogenic cells and their descendants (streptomycin-resistant) as well as lysogenized susceptible cells (rifampicin-resistant) and their descendents. Each data point represents the mean value of three independent values. Error bars as in Figure 1.

In a structured habitat, the bacterial population marked with streptomycin resistance (the only lysogenic cells in the beginning of the competition assay) have an advantage over rifampicin-resistant cells (initially susceptible to λ phage) at all initial ratios of lysogenic to susceptible cells (Figure 1B; regression lines with positive slopes; ANOVA, d.f. = (1,10), p<8×10^−3^ for all lines). However, in the structured habitat, most susceptible cells (rifampicin-resistant) became lysogenic (Figure 2), contrary to what happened in the liquid cultures. Figure 3 shows the ratios of lysogenic to susceptible cells during competitions in the structured habitat, already taking into account the lysogenization of rifampicin-resistant cells.

**Figure 4:**
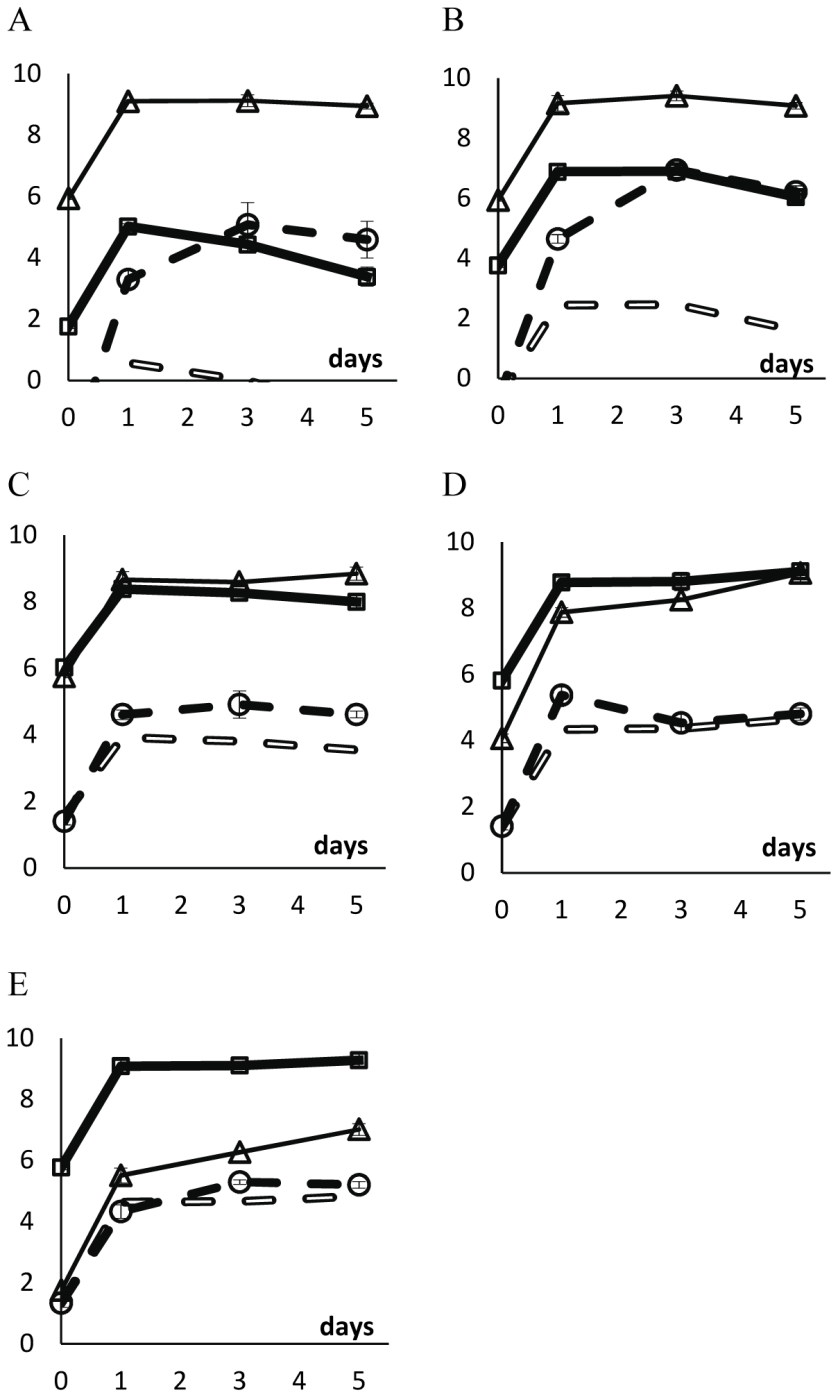
Logarithm of the densities of λ phages and of bacterial strains during competition in the unstructured habitat. Vertical axes represent the log_10_ of the density of lysogenic rifampicin-resistant cells (thick full line with squares), of susceptible streptomycin-resistant cells (thin line with triangles), of lysogenic streptomycin-resistant cells (dotted line), and of the λ phage (filled broken line with circles), in the beginning of competitions (day 0), and after 1, 3 and 5 days of competition. Error bars as in Figure 1. Open broken lines represent the expected density of λ phages if lysogenic cells were the only cells producing the phage; this is calculated assuming that there is a λ phage per 3.13×10^4^ lysogenic cells in pure cultures of lysogenic cells. In all competitions, the initial total bacterial density was around 10^6^ cells ml^−1^. We varied the initial ratio of lysogenic to susceptible cells. A: ratio = 1∶10^4^; B: ratio = 1∶10^2^; C: ratio = 1∶1; D: ratio = 10^2^∶1; E: ratio = 10^4^∶1.

In Figures 4 (unstructured habitat) and 5 (structured habitat), we show the density of the two competing strains as well as the density of free λ phages in the medium. We also show the expected density of λ phages if lysogenic cells were growing in pure cultures. This value was calculated knowing that, in pure cultures of lysogenic cells, there is a free λ phage per (3.13±0.81)×10^4^ lysogenic cells (mean ± twice the standard error). We consider that there is phage amplification among susceptible cells if the density of phages in the competition assay is more than tenfold the typical value found in pure cultures of lysogenic cells, that is, if phage density is 3.13×10^5^ phages per ml or more.

As mentioned above, in the unstructured habitat, the ratios of lysogenic to susceptible cells decreased between day 1 and day 5 (Figure 1A). However, the cause of such decrease is not the same when the initial ratio is low (Figures 4A and 4B) or high (Figures 4D and 4E). When lysogens are initially rare, their density decreases (ANOVA, d.f. = (1,7); ratio of 10^−4^: p<0.0001; ratio of 10^−2^: p<0.01) whereas the density of susceptible cells is stable (ANOVA, d.f. = (1,7), p>0.05). When susceptible cells are initially rare, their density increases (ANOVA d.f. = (1,7); ratio of 10^4^: p<0.00001; ratio of 10^2^: p<0.0001) whereas the density of lysogens is stable (ANOVA, d.f. = (1,7), p>0.05). ). Interestingly, at low initial ratios (10^−4^ and 10^−2^), the virus does not impose a discernible cost to susceptible cells. Presumably this is because the density of viruses is very low and the density of susceptible cells is very high. Nevertheless, at these low initial ratios, susceptible cells do play a role as amplifiers of viruses, as virus density is much higher in these competitions than it would be expected if only lysogenic cells were present in the culture.

When the initial ratios of lysogenic to susceptible cells are 10^0^, 10^2^ and 10^4^, susceptible cells do not amplify the virus (Figures 4C, 4D and 4E). Moreover, when ratios are 10^2^ or 10^4^, susceptible cells are able to increase in density between days 1 and 5 (ANOVA, d.f. = (1,7), p<0.0001). One can explain these observations with the fact that the λ virus is also adsorbed by lysogenic cells (but viruses are not able to replicate inside these cells).

**Figure 5:**
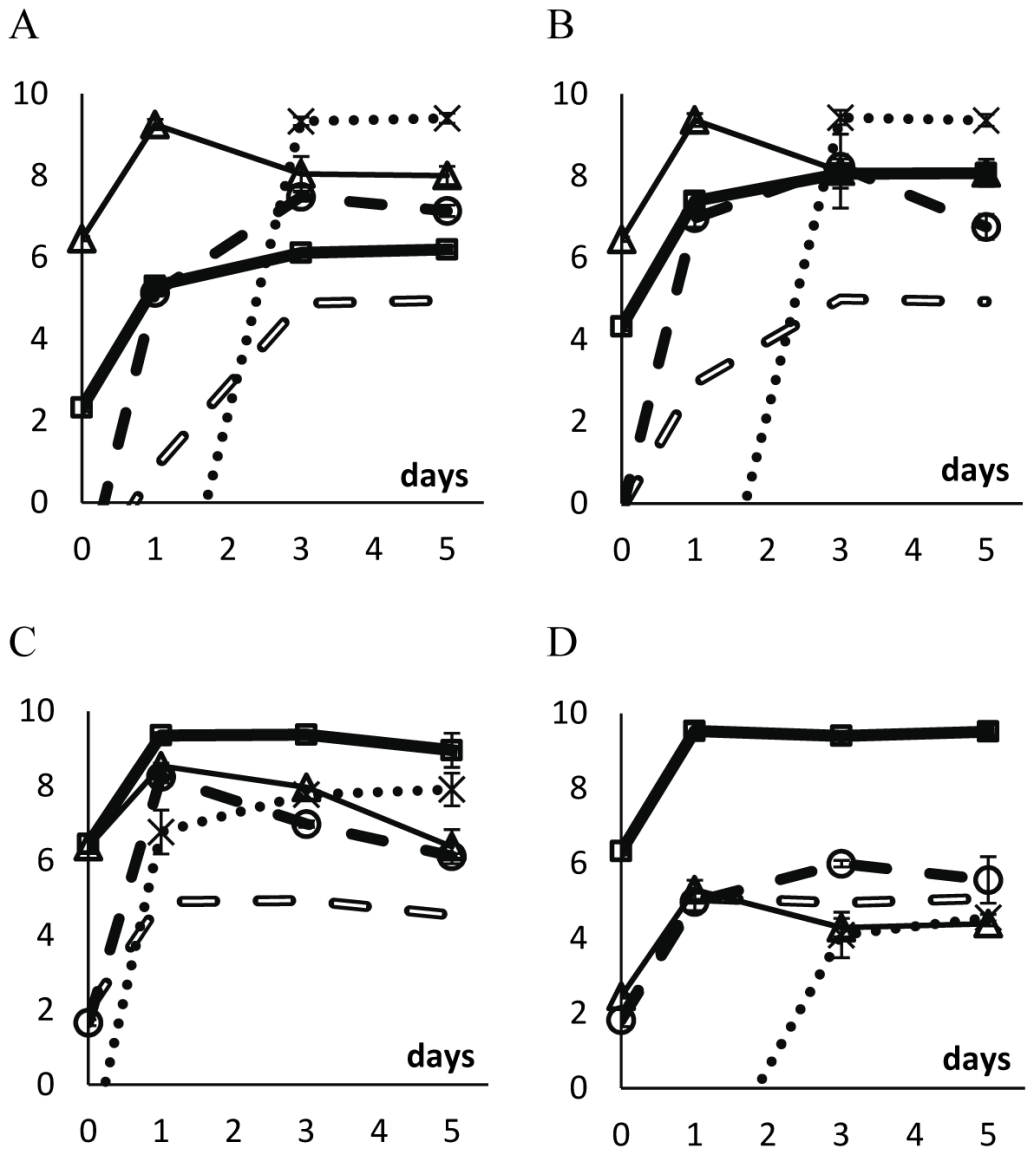
Logarithm of the densities of λ phages and bacterial cells during competitions in the structured habitat. Vertical axes represent the log_10_ of the density of lysogenic rifampicin-resistant cells (thick full line with squares), of susceptible streptomycin-resistant cells (thin line with triangles), of lysogenic streptomycin-resistant cells (dotted line), and of the λ phage (filled broken line with circles), in the beginning of competitions (day 0), and after 1, 3 and 5 days of competition. Error bars as in Figure 1. Open broken lines represent the expected density of λ phages if lysogenic cells were the only cells producing the phage; this is calculated assuming that there is a λ phage per 3.13×10^4^ lysogenic cells in pure cultures of lysogenic cells. In all competitions, the initial total bacterial density was around 3×10^6^ cells ml^−1^ of soft LB agar medium. We varied the initial ratio of lysogenic to susceptible cells. A: ratio = 1∶10^4^; B: ratio = 1∶10^2^; C: ratio = 1∶1; D: ratio = 10^4^∶1.

In competitions performed in soft agar plates, there is virus amplification in the competition assays in which the ratio of lysogenic to susceptible cells is initially close to 10^−4^, 10^−2^, or 10^0^ (Figures 5A, 5B and 5C). Indeed, in these competition experiments, and between days 1 and 5, the density of free viruses is higher than tenfold the density of viruses when lysogenic cells are growing in a pure culture (i.e., in the absence of susceptible cells). However, there is no amplification of viruses by susceptible cells when the ratio of lysogenic to susceptible cells is initially 10^4^ (Figure 5D). This was expected given that, in these competitions (Figure 5D) the density of susceptible cells is much lower than in the previously mentioned competitions (Figures 5A, 5B and 5C).

In the structured habitat, the density of susceptible cells decreases at all initial ratios (ANOVA d.f. = (1,7); ratio of 10^−4^: p<0.01; ratio of 10^−2^: p<0.05; ratio of 10^0^: p<0.0004; ratio of 10^4^: p<0.02). The density of lysogenic cells, however, increases when initially rare (Figure 5A and 5B; ANOVA d.f. = (1,7); ratio of 10^−4^: p<0.003; ratio of 10^−2^: p<0.005) and is stable when ratios are 10^0^ and 10^4^ (Figure 5C and 5D; ANOVA d.f. = (1,7); p>0.05).

**Figure 6:**
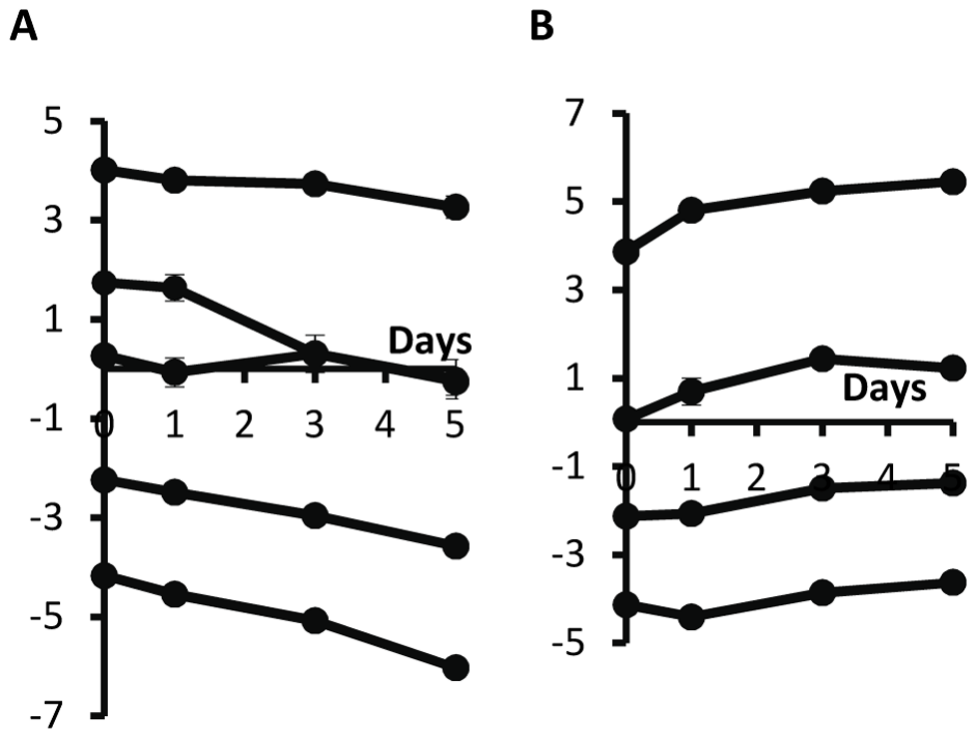
Logarithm of the ratio of expected densities of lysogenic streptomycin-resistant to λ-resistant rifampicin-resistant cells (A) in unstructured and (B) in structured environments. Error bars as in Figure 1.

In order to isolate the effect of λ phage in competition experiments, we reasoned as follows. Two main factors may have an impact on the values of the Str^R^/Rif^R^ ratios: the λ phage and chromosomal markers. Therefore, we performed competition experiments, this time where rifampicin-resistant cells are resistant to the λ phage. In these experiments, the initial ratios Str^R^/Rif^R^ were 10^−6^, 10^−5^,…, 10^6^. The ratios of the density of each strain were measured for periods of 24 and 48 hours (the same time periods used in Figure 1). Then, we calculated the expected density of lysogenic cells if antibiotic-resistance markers had no effect (see Material and Methods S1 for a mathematical explanation). This was possible because we compared the growth rates of these three strains (lysogenic (Str^R^), λ-susceptible (Rif^R^) and λ-resistant (Rif^R^)) and they are similar, as stated before. With this method we isolated the effect of λ phage during competitions. By comparing Figure 1 with Figure 6 one can see that, while these markers indeed affect our competition assays, they do not change our major conclusions.

As just mentioned, the distinction between the strains involved in the competition experiments was possible due to genetic markers (at *rpoB* and *rpsL* genes) conferring antibiotic resistance. So, what would happen if competition experiments were performed with reverted markers?

Unfortunately, mutations in the *rpoB* and *rpsL* genes can affect the viral development of λ phage. Different mutations in the *rpo*B gene have distinguished effects on the phage development (for a review, see ref. [41]). For example, mutation *rpo*B111 [42] restricts the growth of phage λ, while mutation *rpo*B501 [43] decreases the frequency of lysogenization and lysis is delayed because the endolysin synthesis is reduced. Mutations in the *rps*L gene (encoding the ribosomal protein S12) can restrict the synthesis of viral proteins and/or decrease the efficiency of translational initiation, even though the degree of restriction varies according to the mutation [44], [45]. Therefore, the interactions when a given rifampicin-resistant cell is the lysogen may be different from the interactions when the rifampicin-resistant strain is the recipient of the lambda phage (susceptible host). The same reasoning applies for streptomycin-resistant cells. Therefore, it is not certain that, in competition experiments, a rifampicin-resistance lysogen behaves exactly like a streptomycin-resistant lysogen. Similarly, it is not certain that a rifampicin-resistant susceptible host cell behaves like a streptomycin-resistant host cell. The implication of this is that one may observe a higher or lower level of phage amplification and/or lysogenization rate with other chromosomal markers – which includes the situation in which the markers are reversed, as is explained above. In a wider perspective, and given previous works concerning the study of epistasis when mutations at the *rpoB* and *rpsL* are involved [46], [47], [48], epistasis between these mutations and λ-phage are indeed expected. Because of these effects, competition experiments between lysogenic and susceptible strains with reverted markers are not expected to give the same result as with the original markers. To check this, we performed competition experiments for initial ratios of 10^−4^ and 10^4^ in structured and unstructured media. Before that, however, we compared the fitness of the strains that were constructed to performed competitions (with original and reverted markers), and we did not find significant differences (one-way ANOVA, d.f. = (3,16), p = 0.06). Then, with reverted markers, we performed competitions between lysogens and susceptible strains.

**Figure 7:**
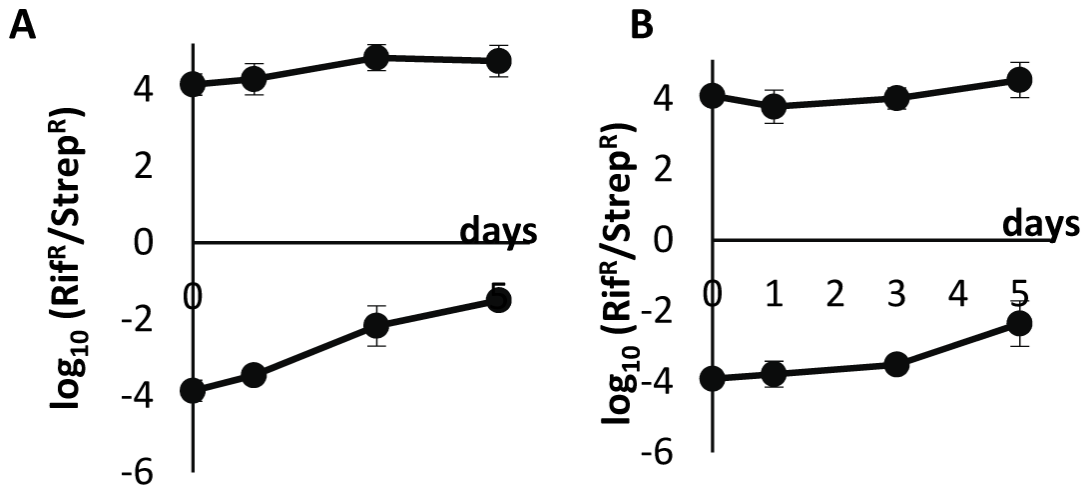
The same as Figure 1, this time with exchanged markers. Logarithm of the ratio of rifampicin-resistant to streptomycin-resistant strains during competitions (A) in unstructured and (B) in structured environments. In the beginning of competitions, rifampicin-resistant cells were lysogenic and streptomycin-resistant cells were susceptible to λ phage. In both habitats, initial ratios were 1∶10^4^, and 10^4^∶1 Each data point represents the mean value of three independent competitions. Error bars as in Figure 1.

In Figure 7A one can see that there was no disadvantage for lysogenic cells in the unstructured environment (contrary to what we observe in Figure 1A). Since the fitnesses of the strains are similar, differences between Figures 1A and 7A are not due to costs imposed by the antibiotic-resistance markers *per se*, but by interactions between the host mutations and the phage genetic system.

In Figure 7B, one can see that, in structured habitat, results obtained with reverted markers are qualitatively similar to the ones obtained previously (Figure 1B).

**Figure 8:**
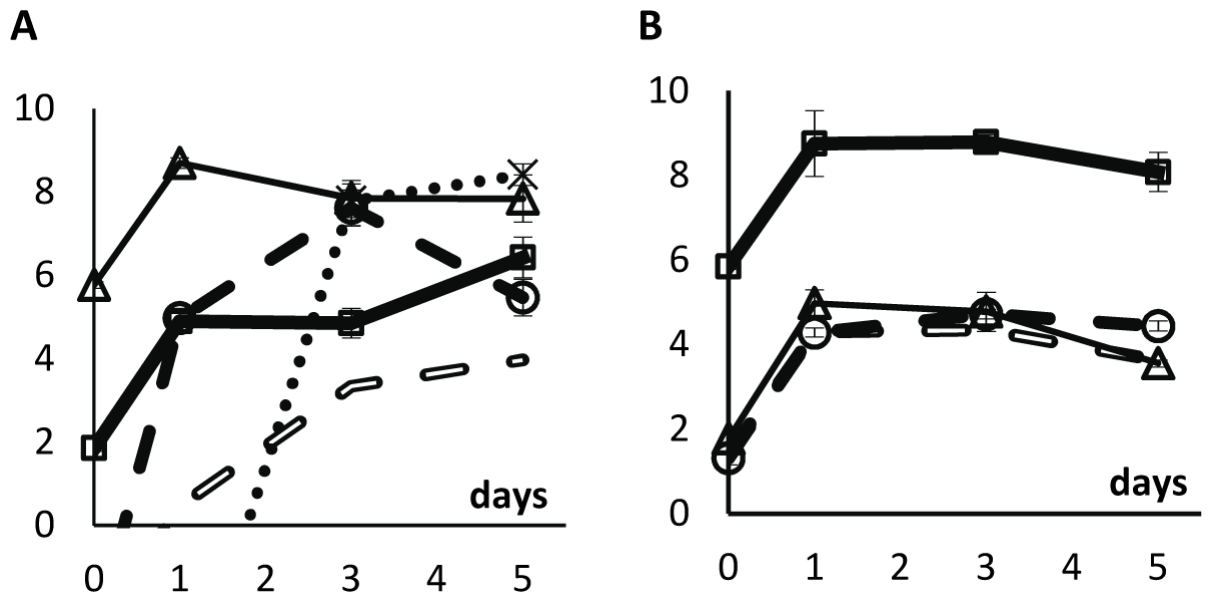
Logarithm of the densities of the λ phage and bacterial strains populations during competition in the unstructured habitat with reversed markers. Vertical axes represent the log_10_ of the density of lysogenic rifampicin-resistant cells (thick full line with squares), of susceptible streptomycin-resistant cells (thin line with triangles), of lysogenic streptomycin-resistant cells (dotted line), and of the λ phage (filled broken line with circles), in the beginning of competitions (day 0), and after 1, 3 and 5 days of competition. Many streptomycin-resistant cells became λ-resistant already at days 3 and 5 (data not shown) in this competition with densities similar to the ones reached by lysogenic streptomycin-resistant cells. Error bars as in Figure 1. Open broken lines represent the expected density of λ phages if lysogenic cells were the only cells producing the phage; this is calculated assuming that there is a λ phage per 3.13×10^4^ lysogenic cells in pure cultures of lysogenic cells. In all competitions, the initial total bacterial density was around 10^6^ cells ml^−1^. A: initial ratio of lysogenic to susceptible cells = 1∶10^4^; B: initial ratio of lysogenic to susceptible cells = 10^4^∶1.

**Figure 9:**
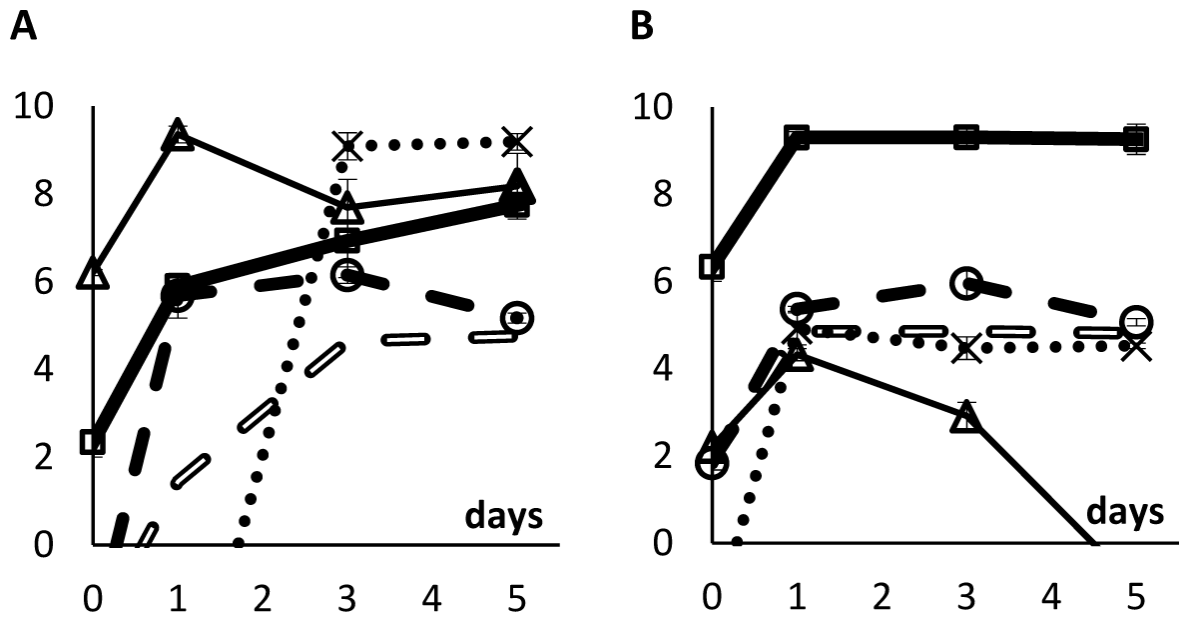
Logarithm of the densities of λ phages and of bacterial strains during competition in the structured habitat with reversed markers. Vertical axes represent the log_10_ of the density of lysogenic rifampicin-resistant cells (thick full line with squares), of susceptible streptomycin-resistant cells (thin line with triangles), of lysogenic streptomycin-resistant cells (dotted line), and of the λ phage (filled broken line with circles), in the beginning of competitions (day 0), and after 1, 3 and 5 days of competition. Error bars as in Figure 1. Open broken lines represent the expected density of λ phages if lysogenic cells were the only cells producing the phage; this is calculated assuming that there is a λ phage per 3.13×10^4^ lysogenic cells in pure cultures of lysogenic cells. In all competitions, the initial total bacterial density was around 10^6^ cells ml^−1^. A: initial ratio of lysogenic to susceptible cells = 1∶10^4^; B: initial ratio of lysogenic to susceptible cells = 10^4^∶1.

Figures 8 and 9 show more details of competitions performed in unstructured and structured media with reverted markers. In structured media results with original and reverted markers are similar (compare Figures 5 and 9), as well as in liquid when the initial frequency is 10^4^ (compare Figures 4E and 8B). However, in liquid media, when the initial frequency was 10^−4^, results are considerably different, because (i) the appearance of lysogens among the initial susceptible cells (near 10^8^ lysogens per ml after 3 days only, Figure 8A), which did not happen with original markers (Figure 4A); (ii) therefore, levels of phage in the medium are much higher (compare Figures 4A and 8A); (iii) hence selecting for the appearance of λ-resistant and lysogenic cells among the susceptible streptomycin-resistant cells (near 10^8^ lysogens per ml after 3 days only, something that did not happen with original markers).

## Discussion

In unstructured media, secreted molecules from a few bacterial cells are expected to be equally spread all over the liquid volume, which may imply that the secreted molecule is at low concentration in the full volume. However, in structured media, excreted molecules are likely to be concentrated in the vicinity of producer cells and to be almost totally absent in the rest of the media.

Now suppose that the excreted substance has an antagonistic effect (e.g., a toxin). If higher concentrations of these molecules imply higher antagonistic effects, the antagonistic effect is predicted to be higher in structured than in unstructured habitats simply because, in this case, the concentration of the molecule is high in the neighbourhood of producers. Therefore, unless the production of toxins is extremely high, one may expect that the outcome of competition experiments between toxin producers and sensitive cells is frequency dependent in which producer cells are at an advantage only when fairly common [24].

If the “toxic substance” is a temperate bacteriophage, the dynamics of the system becomes more interesting because the virus may replicate inside their victims just before killing them [14], [15]. However, the outcome of competition is more difficult to predict than in the case of toxins because, with temperate phages, there is the possibility of lysogenization. Finally, at least in the case of λ phage, predictions are even more complicated because there is a positive correlation between λ phage concentration in the medium and the probability of lysogenization [49], [50], [51].

Given these opposing forces, we asked whether λ phages can be used by their hosts as biological weapons as an alternative to toxins. For that, competition experiments between lysogenic cells and susceptible cells were performed.

In liquid cultures there is phage-amplification when lysogens are initially rare: this observation arises from the fact that there are many more phages in the medium than the number of expected phages if lysogens were alone (Figures 4A, 4B, and 8A). In liquid culture, with reverted chromosomal markers, we also observed lysogenization at days 3–5 (Fig. 8A) – probably because, already at day 1, the density of phages was 100-fold higher with reverted markers (around10^5^ phages/ml) than with the original markers (around 10^3^ phages/ml). It is interesting to note these differences with different markers as they suggest the presence of epistasis between λ-genes and the chromosomal markers at *rpsL* or *rpoB* genes.

In the structured habitat there is phage amplification in most conditions (Fig. 5 and Fig. 9), and most susceptible cells became lysogenic, hence immune to the phage (Fig. 5 and Fig. 9).

Given these results we conclude that bacteriophages indeed behave as allelopathic agents because the susceptible population can suffer from the impact of phages in both types of habitat. However, this is a transient advantage because, in our experiments, lysogenization of susceptible cells occurs rapidly. This would not be possible with toxins (unless, of course, they were coded in mobile elements such as conjugative plasmids). In other words, although phages act as efficient allelopathic agents, the immunity genes are also offered to competitors through lysogenization (with the exception of low frequency of lysogens in liquid media where amplification was not followed by lysogenization).

As explained above, one should certainly expect higher levels of lysogenization in structured habitats than in a liquid environment because lysogeny is favoured when many phages enter into the same cell at once [49], [50], [51]. Indeed, while in unstructured environments, the ratio of the density of λ phages to bacterial cells usually takes a very low value, in structured environments, the proportion of lysogenic to susceptible cells at a local scale may be radically different from the value at a global scale. Therefore, in the context of a structured habitat, one has to consider local interactions.

Some previous studies report similar experiments on both structured habitats and unstructured habitats. Bossi and colleagues tested the contribution of gifsy-1 and -2 viruses to the dynamics of *Salmonella enterica* serovar Typhimurium bacterial populations. The authors performed competitions in static liquid cultures between infected and uninfected bacterial cells. They showed that spontaneous virus induction in a few lysogenic cells enhanced the competitive fitness of the lysogen population as a whole [52]. Similarly, and using competitions in liquid cultures, Joo et al. showed that *Bordetella bronchiseptica* bacterial cells use BPP-1 temperate viruses to mediate competition with other *B. bronchiseptica* cells [18]. Later, Erickson et al. observed that archived cultures of *S. enterica* serovar Typhimurium in competition in soft agar plates gained some selective advantage over non-archived cultures of *S. enterica* because a small proportion of archived bacterial cells yielded the virus fels-1 and -2 which lysed non-archived cultures of *S. enterica* [53]. In 2006, Brown et al. compared the impact of the φ80 temperate viruses with that of toxins (bacteriocins) in liquid cultures and found that the former behaved as “replicating toxins” for three days. By performing experiments with different ratios of lysogenic to susceptible cells, the authors have shown that, in an unstructured habitat, bacteriophage φ80 is mostly useful in the context of invader offense (that is, when lysogens compete with a resident bacterial community), rather than chemical toxins (bacteriocins) that are favoured in the context of resident defence [15].

Some of these previous works indeed show that some viruses seem to be used by bacterial populations as functional equivalents to toxins. However, while most toxins are not expected to be effective in liquid media if toxin-producers are rare, bacteriophages may be efficient weapons (Fig. 4, Fig. 8 and refs. [15], [18]). Interestingly, it is precisely phage amplification that solves this apparent paradox. In unstructured habitats, each lysogenic cell may interact with most susceptible cells in the liquid medium. If there is amplification of the phage (something impossible with a toxin coded in the chromosome or in a non-mobilizable plasmid) and burst size is 10^2^, then five rounds of phage infection and replication would be enough to reach 10^10^ phages, hence killing most or all susceptible cells [14]. Most likely, this is what happened in previous works performed in unstructured habitats [15], [18] and partly in our results (Fig. 4B and Fig. 4A).

The experiments shown here were performed with the PaPa strain of λ phage, which lacks side tail fibers [54]. Due to this characteristic, this phage propagates better in structured environments than strains with side tail fibers, such as the Ur-λ [55]. Therefore it would be interesting to perform similar experiments with Ur-λ phages because they adsorb better to bacterial cells than λ-PaPa phages, despite having less ability to diffuse in structured environments – hence, Ur-λ phages propagate better in liquid than in structured habitats [55].

Though for a few tens of generations only (before massive lysogenization occurs), we conclude that, indeed, bacterial cells may use bacteriophages as replicating toxins. Despite the risk that phages join competitor bacterial cells, hence not being trustworthy weapons, they may still be extremely useful because, a few days may be enough for lysogens to invade and establish in a new habitat [15].

## Supporting Information

Material and Methods S1 (DOC)

## References

[1] Duckworth DH (1976) Who Discovered Bacteriophage? Bacteriological Reviews 40: 793–802. 795414 10.1128/br.40.4.793-802.1976 PMC413985

[2] d'Herelle F (1917) An invisible antagonist microbe of dysentery bacillus. Comptes Rendus Hebdomadaires Des Seances De L Academie Des Sciences 165: 373–375.

[3] Twort FW (1915) An investigation on the nature of ultra-microscopic viruses. Lancet 2: 1241–1243. 10.1017/s0022172400043606 PMC2170983 20475326

[4] Summers WC (2006) Phage and the Early Development. In: Calendar R, Abedon ST, editors. The Bacteriophages. 2nd ed. Oxford: Oxford University Press. 3–7.

[5] Campbell AM (1996) Bacteriophages. In: Neidhart FC, others, editors. Escherichia coli and Salmonella - Cellular and Molecular Biology. 2nd Edition ed. Washinghton: American Society for Microbiology.

[6] Oppenheim AB, Kobiler O, Stavans J, Court DL, Adhya S (2005) Switches in bacteriophage lambda development. Annual Review of Genetics 39: 409–429. 10.1146/annurev.genet.39.073003.113656 16285866

[7] Brussow H, Canchaya C, Hardt WD (2004) Phages and the evolution of bacterial pathogens: From genomic rearrangements to lysogenic conversion. Microbiology and Molecular Biology Reviews 68: 560–602. 15353570 10.1128/MMBR.68.3.560-602.2004 PMC515249

[8] Casas V, Maloy S (2011) Role of bacteriophage-encoded exotoxins in the evolution of bacterial pathogens. Future Microbiology 6: 1461–1473. 22122442 10.2217/fmb.11.124

[9] Edlin G, Lin L, Kudrna R (1975) Lambda lysogens of E. coli reproduce more rapidly than non-lysogens. Nature 255: 735–737. 1094307 10.1038/255735a0

[10] Lin L, Bitner R, Edlin G (1977) Increased reproductive fitness of Escherichia coli lambda lysogens. J Virol 21: 554–559. 319255 10.1128/jvi.21.2.554-559.1977 PMC353857

[11] Edlin G, Lin L, Bitner R (1977) Reproductive fitness of P1, P2, and Mu lysogens of Escherichia coli. J Virol 21: 560–564. 319256 10.1128/jvi.21.2.560-564.1977 PMC353858

[12] Rozsa L (1999) Influencing random transmission is a neutral character in hosts. Journal of Parasitology 85: 1032–1035. 10647033

[13] Rozsa L (2000) Spite, xenophobia, and collaboration between hosts and parasites. Oikos 91: 396–400.

[14] Dionisio F (2007) Selfish and spiteful behaviour through parasites and pathogens. Evolutionary Ecology Research 9: 1199–1210.

[15] Brown SP, Le Chat L, De Paepe M, Taddei F (2006) Ecology of microbial invasions: Amplification allows virus carriers to invade more rapidly when rare. Current Biology 16: 2048–2052. 17055985 10.1016/j.cub.2006.08.089

[16] Brown SP, Inglis RF, Taddei F (2009) Evolutionary ecology of microbial wars: within-host competition and (incidental) virulence. Evolutionary Applications 2: 32–39. 25567845 10.1111/j.1752-4571.2008.00059.x PMC3352407

[17] Brown SP, West SA, Diggle SP, Griffin AS (2009) Social evolution in micro-organisms and a Trojan horse approach to medical intervention strategies. Philosophical Transactions of the Royal Society B-Biological Sciences 364: 3157–3168. 10.1098/rstb.2009.0055 PMC2781867 19805424

[18] Joo J, Gunny M, Cases M, Hudson P, Albert R, et al (2006) Bacteriophage-mediated competition in Bordetella bacteria. Proc Biol Sci 273: 1843–1848. 16790419 10.1098/rspb.2006.3512 PMC1634791

[19] Riley MA, Goldstone CM, Wertz JE, Gordon D (2003) A phylogenetic approach to assessing the targets of microbial warfare. J Evol Biol 16: 690–697. 14632232 10.1046/j.1420-9101.2003.00575.x

[20] Riley MA, Wertz JE (2002) Bacteriocin diversity: ecological and evolutionary perspectives. Biochimie 84: 357–364. 12423779 10.1016/s0300-9084(02)01421-9

[21] Baba T, Schneewind O (1998) Instruments of microbial warfare: bacteriocin synthesis, toxicity and immunity. Trends Microbiol 6: 66–71. 9507641 10.1016/S0966-842X(97)01196-7

[22] Cascales E, Buchanan SK, Duche D, Kleanthous C, Lloubes R, et al (2007) Colicin biology. Microbiol Mol Biol Rev 71: 158–229. 17347522 10.1128/MMBR.00036-06 PMC1847374

[23] Kerr B, Riley MA, Feldman MW, Bohannan BJ (2002) Local dispersal promotes biodiversity in a real-life game of rock-paper-scissors. Nature 418: 171–174. 12110887 10.1038/nature00823

[24] Chao L, Levin BR (1981) Structured Habitats and the Evolution of Anticompetitor Toxins in Bacteria. Proceedings of the National Academy of Sciences of the United States of America-Biological Sciences 78: 6324–6328. 10.1073/pnas.78.10.6324 PMC349031 7031647

[25] West SA, Diggle SP, Buckling A, Gardner A, Griffins AS (2007) The social lives of microbes. Annual Review of Ecology Evolution and Systematics 38: 53–77.

[26] Hamilton WD (1970) Selfish and Spiteful Behaviour in an Evolutionary Model. Nature 228: 1218–&. 10.1038/2281218a0 4395095

[27] Grafen A (1985) A geometric view of relatedness. Oxford Surveys in Evolutionary Biology 2: 28–90.

[28] Foster KR, Ratnieks FLW, Wenseleers T (2000) Spite in social insects. Trends in Ecology & Evolution 15: 469–470.

[29] Gardner A, West SA, Buckling A (2004) Bacteriocins, spite and virulence. Proceedings of the Royal Society of London Series B-Biological Sciences 271: 1529–1535. 10.1098/rspb.2004.2756 PMC1691756 15306326

[30] Gardner A, West SA (2004) Spite and the scale of competition. Journal of Evolutionary Biology 17: 1195–1203. 15525404 10.1111/j.1420-9101.2004.00775.x

[31] Queller DC (1994) Genetic Relatedness in Viscous Populations. Evolutionary Ecology 8: 70–73.

[32] West SA, Pen I, Griffin AS (2002) Conflict and cooperation - Cooperation and competition between relatives. Science 296: 72–75. 11935015 10.1126/science.1065507

[33] Iwasa Y, Nakamaru M, Levin SA (1998) Allelopathy of bacteria in a lattice population: Competition between colicin-sensitive and colicin-producing strains. Evolutionary Ecology 12: 785–802.

[34] Wiener P (2000) Antibiotic production in a spatially structured environment. Ecology Letters 3: 122–130.

[35] Gordon DM (1992) Rate of Plasmid Transfer among Escherichia-Coli Strains Isolated from Natural-Populations. Journal of General Microbiology 138: 17–21. 1556548 10.1099/00221287-138-1-17

[36] Dionisio F, Matic I, Radman M, Rodrigues OR, Taddei F (2002) Plasmids spread very fast in heterogeneous bacterial communities. Genetics 162: 1525–1532. 12524329 10.1093/genetics/162.4.1525 PMC1462386

[37] Frank SA (1994) Spatial Polymorphism of Bacteriocins and Other Allelopathic Traits. Evolutionary Ecology 8: 369–386.

[38] Chumpolkulwong N, Hori-Takemoto C, Hosaka T, Inaoka T, Kigawa T, et al (2004) Effects of Escherichia coli ribosomal protein S12 mutations on cell-free protein synthesis. Eur J Biochem 271: 1127–1134. 15009191 10.1111/j.1432-1033.2004.04016.x

[39] Death A, Notley L, Ferenci T (1993) Derepression of LamB protein facilitates outer membrane permeation of carbohydrates into Escherichia coli under conditions of nutrient stress. J Bacteriol 175: 1475–1483. 8444809 10.1128/jb.175.5.1475-1483.1993 PMC193235

[40] Pelosi L, Kuhn L, Guetta D, Garin J, Geiselmann J, et al (2006) Parallel changes in global protein profiles during long-term experimental evolution in Escherichia coli. Genetics 173: 1851–1869. 16702438 10.1534/genetics.105.049619 PMC1569701

[41] Friedman DI, Olson ER, Georgopoulos C, Tilly K, Herskowitz I, et al (1984) Interactions of bacteriophage and host macromolecules in the growth of bacteriophage lambda. Microbiol Rev 48: 299–325. 6240590 10.1128/mr.48.4.299-325.1984 PMC373221

[42] Jin DJ, Gross CA (1989) Characterization of the pleiotropic phenotypes of rifampin-resistant rpoB mutants of Escherichia coli. J Bacteriol 171: 5229–5231. 2670912 10.1128/jb.171.9.5229-5231.1989 PMC210350

[43] Lecocq J, Dambly C (1976) A bacterial RNA polymerase mutant that renders lambda growth independent of the N and cro functions at 42 degrees C. Mol Gen Genet. 145: 53–64. 10.1007/BF00331557 775309

[44] Yates JL, Gette WR, Furth ME, Nomura M (1977) Effects of ribosomal mutations on the read-through of a chain termination signal: studies on the synthesis of bacteriophage lambda O gene protein in vitro. Proc Natl Acad Sci U S A 74: 689–693. 322139 10.1073/pnas.74.2.689 PMC392358

[45] Li MY, Weng ML, Tong KZ (1998) Mechanism of regulating the expression of lambda N gene by ribosomal protein at translational level. Science in China Series C-Life Sciences 41: 29–36. 10.1007/BF02882703 18726268

[46] Trindade S, Sousa A, Xavier KB, Dionisio F, Ferreira MG, et al (2009) Positive Epistasis Drives the Acquisition of Multidrug Resistance. Plos Genetics 5: e1000578. 19629166 10.1371/journal.pgen.1000578 PMC2706973

[47] Silva RF, Mendonca SCM, Carvalho LM, Reis AM, Gordo I, et al (2011) Pervasive Sign Epistasis between Conjugative Plasmids and Drug-Resistance Chromosomal Mutations. PLoS Genetics 7: e1002181. 21829372 10.1371/journal.pgen.1002181 PMC3145620

[48] Breen MS, Kemena C, Vlasov PK, Notredame C, Kondrashov FA (2012) Epistasis as the primary factor in molecular evolution. Nature 490: 535–+. 10.1038/nature11510 23064225

[49] Kourilsky P (1973) Lysogenization by Bacteriophage-Lambda.1. Multiple Infection and Lysogenic Response. Molecular & General Genetics 122: 183–195. 4573866 10.1007/BF00435190

[50] Kourilsky P, Knapp A (1974) Lysogenization by Bacteriophage-Lambda.3. Multiplicity Dependent Phenomena Occurring Upon Infection by Lambda. Biochimie 56: 1517–1523. 4619342

[51] Zeng LY, Skinner SO, Zong CH, Sippy J, Feiss M, et al (2010) Decision Making at a Subcellular Level Determines the Outcome of Bacteriophage Infection. Cell 141: 682–691. 20478257 10.1016/j.cell.2010.03.034 PMC2873970

[52] Bossi L, Fuentes JA, Mora G, Figueroa-Bossi N (2003) Prophage contribution to bacterial population dynamics. J Bacteriol 185: 6467–6471. 14563883 10.1128/JB.185.21.6467-6471.2003 PMC219396

[53] Erickson M, Newman D, Helm RA, Dino A, Calcutt M, et al (2009) Competition among isolates of Salmonella enterica ssp. enterica serovar Typhimurium: role of prophage/phage in archived cultures. FEMS Microbiol Lett 294: 37–44. 19493006 10.1111/j.1574-6968.2009.01554.x

[54] Hendrix RW, Duda RL (1992) Bacteriophage-Lambda Papa - Not the Mother of All Lambda-Phages. Science 258: 1145–1148. 1439823 10.1126/science.1439823

[55] Gallet R, Shao YP, Wang IN (2009) High adsorption rate is detrimental to bacteriophage fitness in a biofilm-like environment. Bmc Evolutionary Biology 9. 10.1186/1471-2148-9-241 PMC2762979 19804637

