# Supplementary material for: Temperate Bacterial Viruses as Double-Edged Swords in Bacterial Warfare
Source: PLoS One. 2013 Mar 11;8(3):e59043. doi: 10.1371/journal.pone.0059043 (PMC3594171; doi:10.1371/journal.pone.0059043)
Supplement: Material and Methods S1 — (DOC) [file pone.0059043.s001.doc]

**Temperate Bacterial Viruses as Double-Edged Swords in Bacterial Warfare**

João Alves Gama, Ana Maria Reis, Iolanda Domingues, Helena Mendes-Soares, Ana Margarida Matos, and Francisco Dionisio

**SUPPORTING MATERIAL AND METHODS S1**

### Isolating the effect of λ-phage in competition experiments

As explained in the main text, we performed competitions between lysogenic (Str^R^) and λ-resistant (Rif^R^) strains. The ratios Str^R^/Rif^R^ were 1:10^6^, 1:10^5^,…, 10^6^:1. The ratios of the density of each strain resulting from these competitions were measured after 24 and 48 hours. Then, we calculated the expected density of lysogenic cells if antibiotic-resistance markers had no effect.

Let $\omega_{A/B}$be the fitness of strain A relative to strain B:

$$\omega_{A/B}=\frac{\log\left(\frac{A_{f}}{A_{i}}\right)}{\log\left(\frac{B_{f}}{B_{i}}\right)}$$

Now, let$\omega_{\lambda^{+},Str^{R}/\lambda^{S},Rif^{R}}$be the fitnesses of lysogenic cells (which are Str^R^) relative to susceptible cells (Rif^R^) and $\omega_{\lambda^{+},Str^{R}/\lambda^{R},Rif^{R}}$be the fitness of lysogenic cells (Str^R^) relative to λ-resistant cells (Rif^R^). Then, to first order, one can calculate the fitness of lysogenic cells relative to isogenic susceptible cells (they are both Rif^R^) as:

$\omega_{\lambda^{+}/\lambda^{S}}$=$\omega_{\lambda^{+},Str^{R}/\lambda^{S},Rif^{R}}$/$\omega_{\lambda^{+},Str^{R}/\lambda^{R},Rif^{R}}$ (Equation S1)

The term $\omega_{\lambda^{+},Str^{R}/\lambda^{S},Rif^{R}}$is the actual value of the competitions shown in Figure 1. As explained above, we measured$\omega_{\lambda^{+},Str^{R}/\lambda^{R},Rif^{R}}$. We measured these values for intervals of one day and intervals of two days because in competition experiments (Fig.1-5), intervals between measurements are of one or two days. We measured these values by performing competition experiments between lysogenic (Str^R^) and λ-resistant (Rif^R^) strains for intervals of one day and two days. With this process, we obtained an array of values of $\omega_{\lambda^{+}/\lambda^{S}(1day)}$and $\omega_{\lambda^{+}/\lambda^{S}(2days)}$.

If we had performed direct competitions between lysogenic cells and susceptible cells (that is, with no chromosomal markers), the fitness $\omega_{\lambda^{+}/\lambda^{S}}$would be calculated as:

$\omega_{\lambda^{+}/\lambda^{S}}=\frac{\log\left(\frac{L_{f}}{L_{i}}\right)}{\log\left(\frac{S_{f}}{S_{i}}\right)}$ (Equation S2)

Note that the values for $\omega_{\lambda^{+}/\lambda^{S}}$, are $\omega_{\lambda^{+}/\lambda^{S}(1day)}$and $\omega_{\lambda^{+}/\lambda^{S}(2days)}$. Therefore, putting together Equations S1 and S2, the density of lysogens after competition would be given by:

$L_{f}=L_{i}\left(\frac{S_{f}}{S_{i}}\right)^{\omega_{\lambda^{+}/\lambda^{S}}}$ (Equation S3)

As a result, L_1(expected)_, L_3(expected)_, and L_5(expected)_ are given by:

$$L_{1(\exp ected)}=L_{0}\left(\frac{S_{1}}{S_{0}}\right)^{\omega_{\lambda^{+}/\lambda^{S}}(1day)}$$

$$L_{3(\exp ected)}=L_{1}\left(\frac{S_{3}}{S_{1}}\right)^{\omega_{\lambda^{+}/\lambda^{S}}(2days)}$$

$$L_{5(\exp ected)}=L_{3}\left(\frac{S_{5}}{S_{3}}\right)^{\omega_{\lambda^{+}/\lambda^{S}}(2days)}$$

One can now plot these values (Figure 6 in the main text).
